# Mps1 is SUMO-modified during the cell cycle

**DOI:** 10.18632/oncotarget.6552

**Published:** 2015-12-10

**Authors:** Agnese Restuccia, Feikun Yang, Changyan Chen, Lou Lu, Wei Dai

**Affiliations:** ^1^ Division of Virus-Associated Carcinogenesis, German Cancer Research Center, Heidelberg, Germany; ^2^ Departments of Environmental Medicine, Biochemistry and Molecular Pharmacology, New York University Langone Medical Center, Tuxedo Park, NY, USA; ^3^ Center for Drug Discovery, Northeastern University, Boston, MA, USA; ^4^ Division of Molecular Medicine, Department of Medicine, David Geffen School of Medicine, University of California Los Angeles, Torrance, CA, USA

**Keywords:** Mps1, mitosis, sumoylation, BubR1

## Abstract

Mps1 is a dual specificity protein kinase that regulates the spindle assembly checkpoint and mediates proper microtubule attachment to chromosomes during mitosis. However, the molecular mechanism that controls Mps1 protein level and its activity during the cell cycle remains unclear. Given that sumoylation plays an important role in mitotic progression, we investigated whether Mps1 was SUMO-modified and whether sumoylation affects its activity in mitosis. Our results showed that Mps1 was sumoylated in both asynchronized and mitotic cell populations. Mps1 was modified by both SUMO-1 and SUMO-2. Our further studies revealed that lysine residues including K71, K287, K367 and K471 were essential for Mps1 sumoylation. Sumoylation appeared to play a role in mediating kinetochore localization of Mps1, thus affecting normal mitotic progression. Furthermore, SUMO-resistant mutants of Mps1 interacted with BubR1 more efficiently than it did with the wild-type control. Combined, our results indicate that Mps1 is SUMO-modified that plays an essential role in regulating Mps1 functions during mitosis.

## INTRODUCTION

The spindle assembly checkpoint (SAC) is a conserved surveillance mechanism that regulates partitioning of duplicated genome into two daughter cells during mitosis [1]. Extensive research in the past has revealed that protein kinases including Aurora A/B, Mps1, Bub1, and BubR1 positively regulate SAC activities, thus arresting cell at metaphase until all condensed chromosomes are correctly orientated [2, 3]. The key molecular target of SAC is Cdc20, a substrate-specific activator of anaphase promoting complex/cyclosome (APC/C) [4]. Cdc20, Mad2, Mad3/BubR1, and Bub3 form the mitotic checkpoint complex (MCC), inhibiting the ubiquitin E3 ligase activity of APC/C [5, 6]. Increasing evidence indicates that SAC components are promising target for cancer drug development [7, 8].

Mps1 (*mono-polar spindle 1*) is an evolutionarily conserved protein that functions as a key component of SAC [9, 10]. Specifically, Mps1 plays an essential role in recruiting Mad1 and Mad2 to unattached kinetochores, thus mediating proper chromosome congression and accurate chromosome segregation [11–17]. Given its essential role in SAC functions, Mps1 undergoes a dynamic distribution during mitosis [10, 18]. Biochemically, Mps1 is phosphorylated on multiple residues and the phosphorylation is essential for its subcellular localization. Threonine 12 (T12) and serine 15 (S15) appear to be critical in mediating the accumulation of Mps1 on kinetochores [19]. Moreover, Mps1 inactivation is at least partly regulated by a proteasome-dependent degradation process mediated by APC^Cdc20^ [20–22]. Timely inactivation of Mps1 is required for normal cell cycle progression as well as the termination of SAC.

Given that sumoylation plays an essential role in regulating the activity of numerous mitotic proteins, we determined whether Mps1 was also modified by sumoylation and whether the modification affected its activity during mitosis. We found that Mps1 was modified by both SUMO-1 and SUMO-2 during the cell cycle. Site-directed mutagenesis coupled with ectopic expression revealed that lysine residues including K71, K287, K367 and K471 were likely SUMO acceptors. Ectopic expression of SUMO-resistant Mps1 resulted in an accelerated mitotic progression. Therefore, our current study reveals a new type of post-translational mechanism that modulates Mps1's function in mitosis.

## RESULTS

### Mps1 is modified by SUMOylation

**Figure 1:**
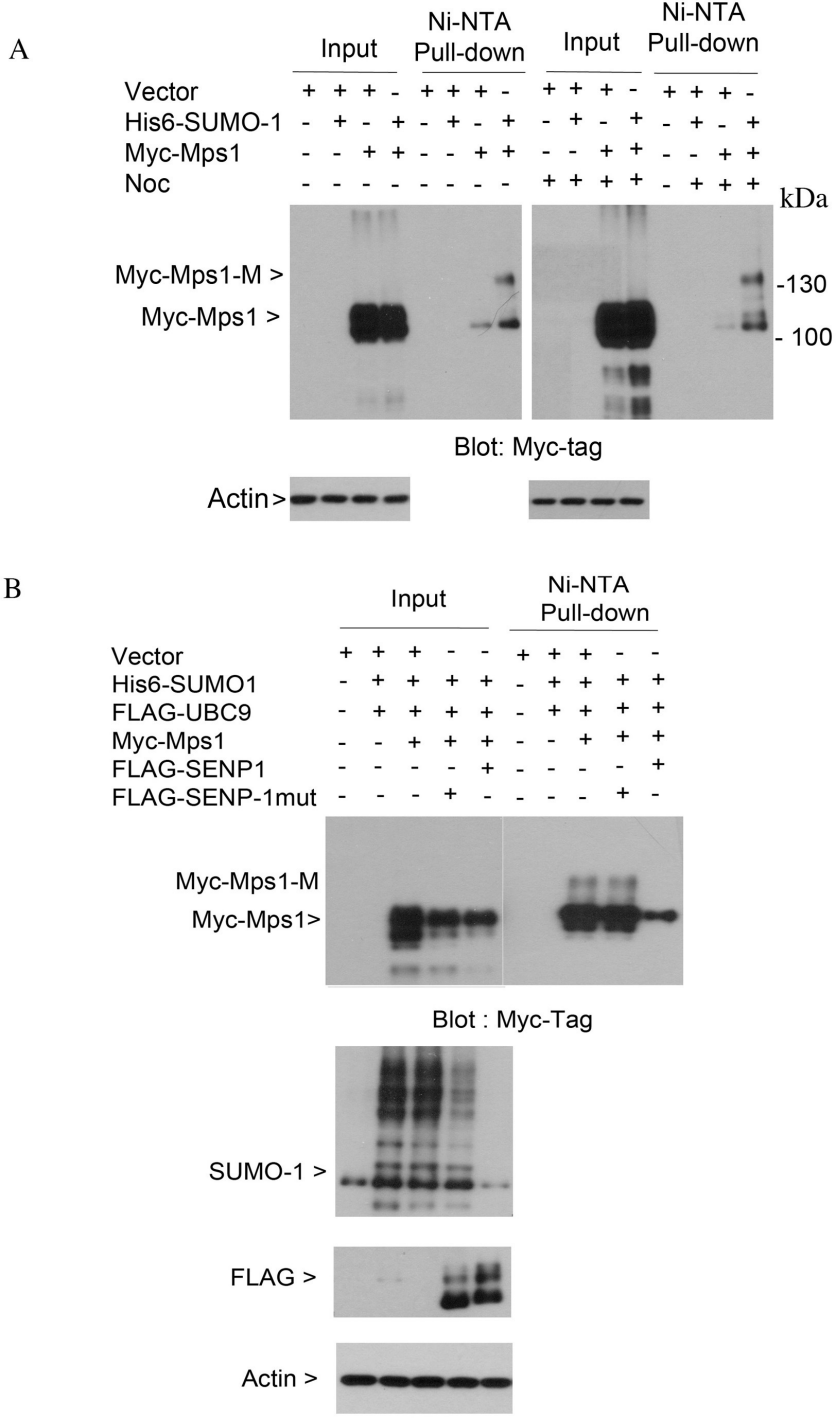
Sumoylation of ectopically expressed Mps1 (**A**) HeLa cells were transfected with plasmids as indicated for 48 h followed by treatment with nocodazole for 16 h. Equal amounts of total cell lysates prepared in the 8 M urea lysis buffer were subject to Ni-IDA pull-down analysis. Protein precipitates were then blotted for Myc-tag. Total lysates were also blotted with the antibody to β-actin. Myc-Mps1-M indicates the modified form of Myc-Mps1. (**B**) HeLa cells were transfected with various plasmids as indicated. Ni-IDA pull-down assay was performed as described in A. Protein precipitates were blotted for Myc-tag. Total lysates were also blotted for SUMO-1, FLAG-tag and β-actin.

To study whether sumoylation plays a direct role in regulating the activity of Mps1 during the cell cycle, we transfected HEK293T cells with a plasmid construct expressing Myc-tagged Mps1, alone or along with plasmids expressing His6-SUMO-1 and FLAG-UBC9. Two days after transfection, cells were treated with nocodazole for 16 h, after which equal amounts of cell lysates prepared under the denature condition were subjected to Ni-IDA affinity pull-down analysis. Precipitated proteins were then blotted with antibodies against Myc-tag. As shown in Figure 1A, slower mobility shift bands were detected exclusively in lysates from cells expressing transfected Myc-Mps1, His6-SUMO-1 and FLAG-UBC9, strongly suggesting the presence of sumoylated Mps1. Moreover, in this transient expression system, the slow mobility shifts were observed in both asynchronized and mitotic cell lysates.

SUMO modification is a reversible process, and de-conjugation of the SUMO moiety from targeted proteins is catalyzed by sentrin-specific isopeptidases (SENPs). To further confirm the slow mobility shifts were sumoylated Mps1, HEK293T cells were transfected with plasmids expressing Myc-tagged Mps1 alone or along with constructs either expressing His6-SUMO-1 and FLAG-tagged wild-type SENP1 or His6-SUMO-1 and FLAG-tagged enzymatically defective SENP1 (SENP1-mut). Forty-eight hours post-transfection, cells were treated with nocodazole for 16 h. Equal amounts of cell lysates prepared under the denature condition were subjected to Ni-IDA affinity pull-down analysis. Precipitated proteins were then blotted with antibodies against the Myc-tag. The slower mobility shifts of Mps1 were completely abolished in cells expressing wild-type SENP1, but not SENP1-mut (Figure 1B, 1st and 2nd panels). Expression of FLAG-SENP1 was confirmed by blotting total lysates with antibodies against the FLAG tag and SUMO1 proteins (Figure 1B, the 3rd and 4th panels). Combined, these results strongly support the notion that ectopically expressed Mps1 can be modified by sumoylation.

### Mps1 is modified by both SUMO-1 and SUMO-2

**Figure 2:**
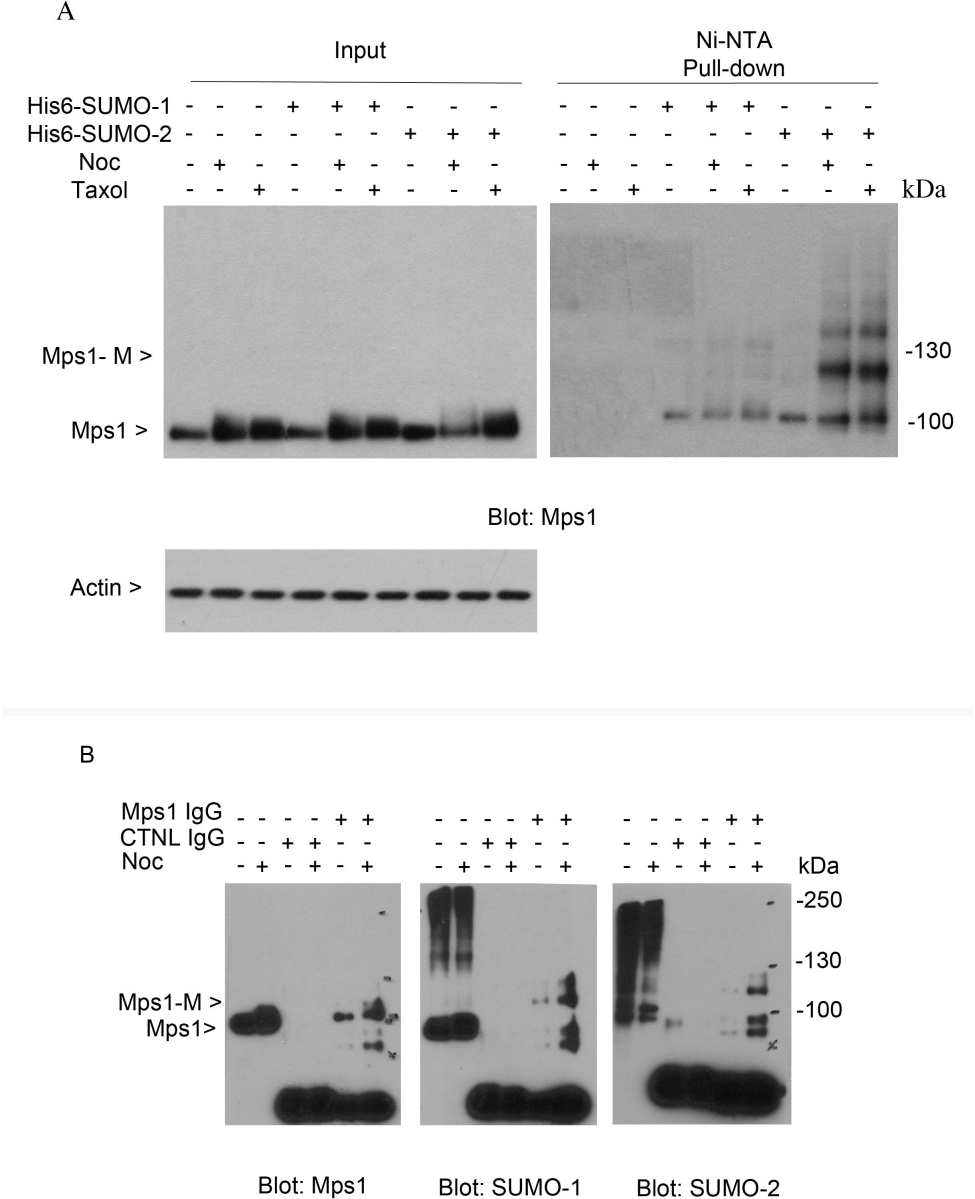
Sumoylation of endogenous Mps1 (**A**) HeLa cells stably expressing His6-SUMO-1 or His6-SUMO-2 proteins were treated with nocodazole or taxol for 16 h, after which cells were lysed and subject to Ni-IDA pull-down assay under denature conditions. Protein precipitates, along with lysate inputs were blotted for Mps1 and β-actin. (**B**) HeLa cells were treated with nocodazole for 16 h, after which cells were lysed and equal amounts of cell lysates prepared in a native condition were immunoprecipitated with Mps1 antibody or a control mouse IgG. Immunoprecipitates were blotted for Mps1, SUMO-1, and SUMO-2. Mps1-M indicates the SUMO modified Mps1.

To understand whether endogenous Mps1 is sumoylated during cell cycle progression, we took advantage of HeLa cell lines that stably expressed His6-tagged SUMO-1 or His6-tagged SUMO-2. Cells were either asynchronized or treated with nocodazole or taxol for 16 h. Equal amounts of cell lysates prepared under a denaturing condition were subjected to Ni-IDA pull-down analysis. Protein precipitates were then blotted with antibodies against Mps1 protein. As shown in Figure 2A, slow mobility bands that were immunoreactive to Mps1 antibodies were detected in both cells expressing His6-SUMO-1 and His6-SUMO-2. Moreover, the signal was enhanced when cells were treated with nocodazole or taxol. These results strongly suggest that endogenous Mps1 is sumoylated during the cell cycle.

To further confirm that endogenous Mps1 is sumoylated, HeLa cells were either asynchronized or treated with nocodazole for 16 h. Equal amount of cell lysates prepared under a native condition were immunoprecipitated with the antibody to Mps1 and the immunoprecipitates were blotted for Mps1, SUMO-1 and SUMO-2. As shown in Figure 2B, both SUMO-1 and SUMO-2 antibodies detected a band that migrated at the same position as the one detected in the pull-down materials by the Mps1 antibody but not by the control IgG. Again, the signal was greatly enhanced when cells were treated with nocodazole. Combined, these results strongly support the notion that endogenous Mps1 is modified by both SUMO-1 and SUMO-2 during the cell cycle.

### Multiple lysine residues are essential for Mps1 SUMOylation

**Figure 3:**
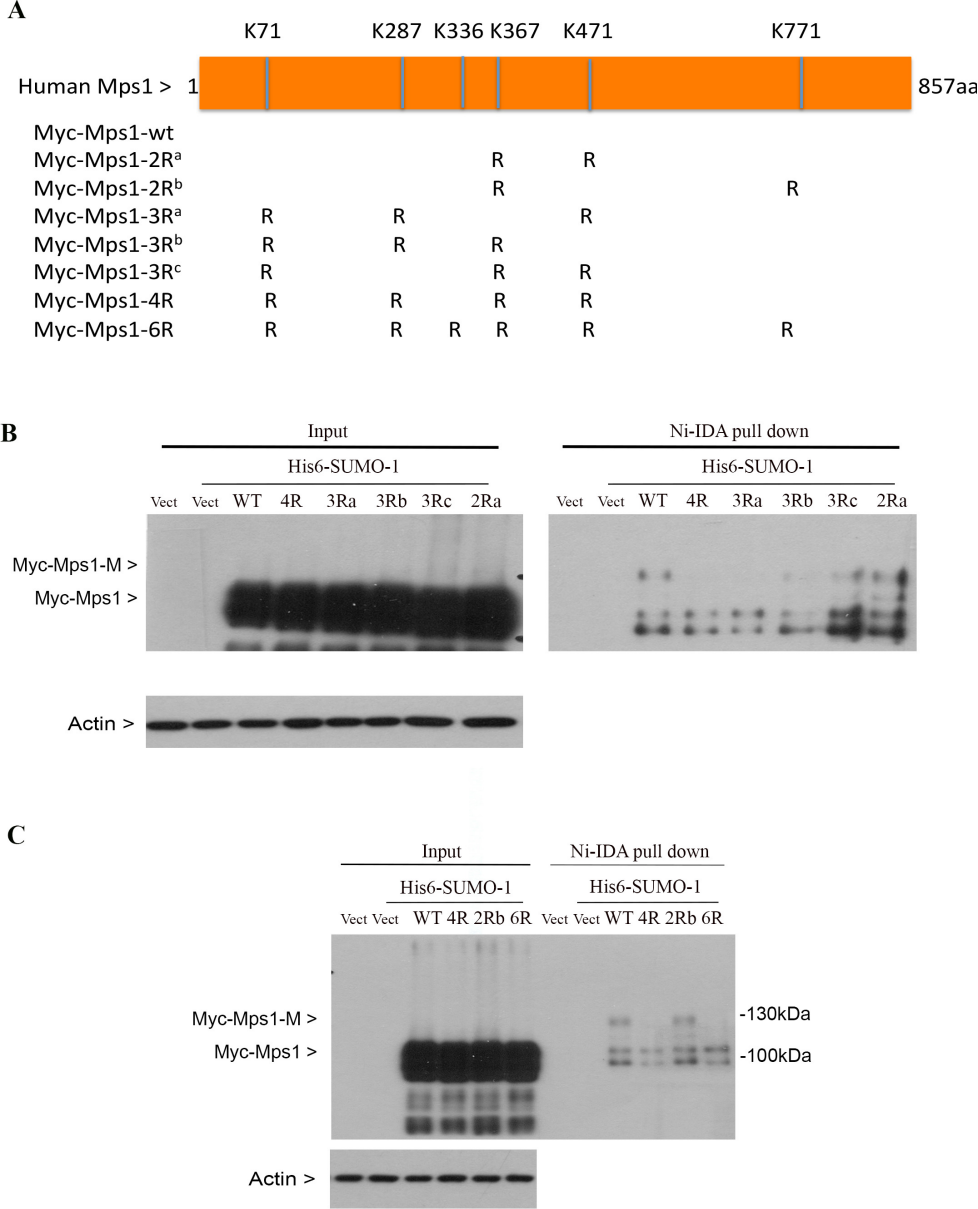
Identification of lysine residues essential for Mps1 sumoylation (**A**) Schematic presentation of human Mps1 wild-type and various mutants used in this study. (**B** and **C**) HEK293T cells were transfected with empty vector (vect) or various Mps1 plasmids together with constructs expressing His6-SUMO-1 protein for 48 h followed by treatment with nocodazole for 16 h. Total cell lysates prepared under denature conditions were subject to Ni-IDA pull-down assay. Protein precipitates, along with lytsate inputs, were blotted for the Myc-tag and β-actin.

To identify the potential lysine residues of sumoylation, we analyzed Mps1 amino acid sequences for optimal sumoylation using the criteria available at Abgent Inc. Six lysines residues (K71, K287, K336, K367, K471, K772) with the highest scores were subjected to mutagenic analysis. Mutant constructs were made that expressed Myc-tagged Mps1 with lysine residues replaced with arginines (Figure 3A). HEK293T were first transfected with indicated Mps1 constructs, along with a plasmid expressing His6-SUMO-1 for 48 h, and then treated with nocodazole for 16 h. Equal amounts of cell lysates prepared with a denaturing buffer were subjected to Ni-IDA pull-down analysis. The precipitates were blotted with the antibody against the Myc-tag. As shown in Figure 3A, wild-type Myc-Mps1 and its mutant constructs were expressed at a comparable level (Figure 3B and 3C, input), suggesting that sumoylation may not affect the protein stability. Importantly, an extra slower mobility band that was immunoreactive to the Myc antibody was detected in cells expressing wild-type or mutant with K336R and K471R but not mutant with all 6 lysine residues replaced with arginies or mutants with 4 lysine residues mutated K71, K287, K367 and K471 (referred as 4R mutants hereafter). Combined, these results indicate that lysines K71, K287, K367 and K471 are essential for Mps1 sumoylation.

### SUMOylation partially affects Mps1 subcellular localization during mitosis

**Figure 4:**
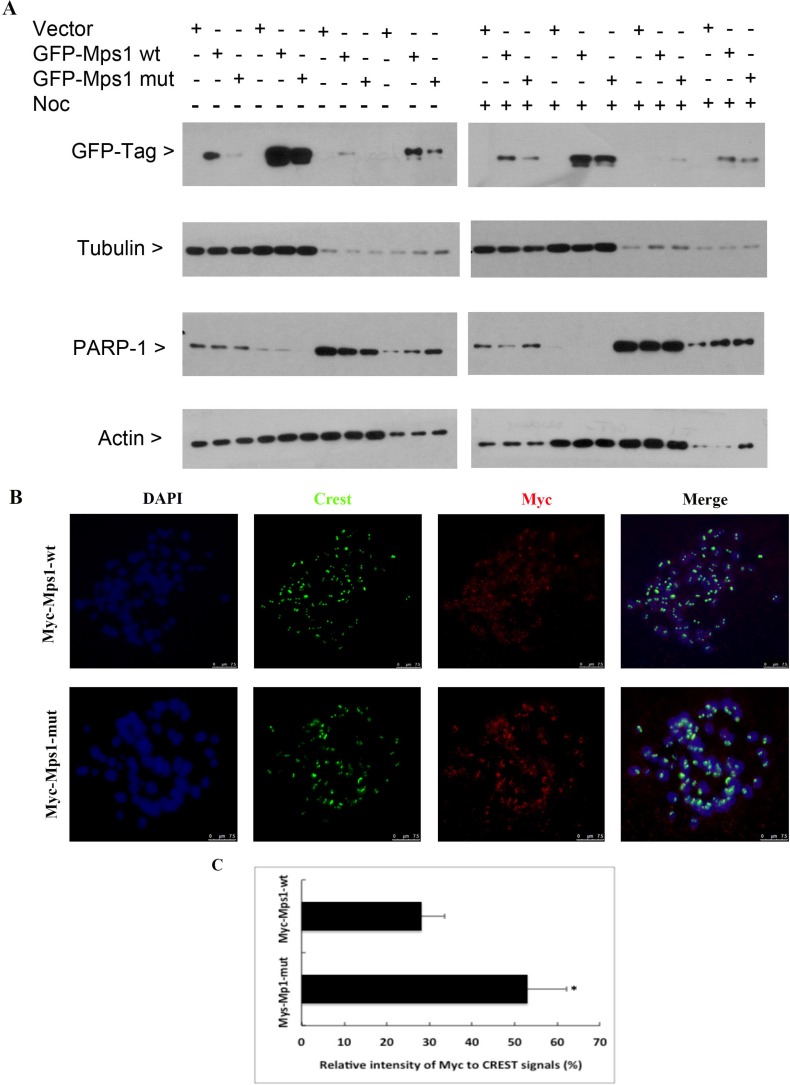
Sumoylation does not significantly affect Mps1 subcellular localization (**A**) HeLa cells were transfected with indicated plasmids for 48 h followed by treatment with nocodazole for 16 h. Cells were then collected for fractionation to isolate cytoplasmic, soluble nuclear, and chromatin binding proteins. Fractionated proteins were then blotted for GFP, α-tubulin, PARP-1 and β-actin. (**B**) HeLa cells were transfected with plasmids expressing Myc-Mps1-wt or Myc-Mps1-mut for 48 h followed by treatment with nocodazole for 4 h prior to chromosome spread analysis. Chromosome spreads were stained overnight with antibodies against Myc-tag and CREST. DNA was counter-stained with DAPI. Representative images show the kinetochore localization of Myc-Mps1. (**C**) The fluorescent intensity of Myc-Mps1 from (B) was analyzed using ImageJ software and normalized to the CREST signal (Myc-Mps1-wt: *n* = 11, Myc-Mps1-mut: *n* = 10).

Sumoylation plays a role in regulating the subcellular localization of target proteins [25–27]. To test whether this is the case for Mps1, HeLa cells were transfected with GFP-tagged wild-type Mps1 (GFP-Mps1-wt) or its SUMO-resistant 4R mutants (GFP-Mps1-mut) for 48 h. Cells were then collected for subcellular fractionation, after which equal amounts of cell lysates were blotted with the antibody against GFP. As shown in Figure 4A (left panel), we did not see apparent subcellular localization difference between GFP-Mps1-wt and its SUMO-resistant counterpart although a relatively lower level of GFP signal was detected in cells transfected with GFP-Mps1-Mut. Next we treated the transfected cells with nocodazole for 16 h. After fractionation, GFP-tag signals were detected by Western blotting. Interestingly, compared with GFP-Mps1-wt, higher levels of GFP-Mps1-mut were detected in the fraction of loose chromatin-binding proteins (Figure 4A, right panel).

Kinetochore localization is required for the mitotic function of Mps1 [28, 29]. To test whether sumoylation regulates Mps1 kinetochore localization, HeLa cells were transfected with a plasmid construct expressing Myc-tagged wild-type Mps1 (Myc-Mps1-wt) or its SUMO-resistant 4R mutant (Myc-Mps1-Mut) for 48 h, followed by treatment with 40 ng/mL nocodazole for 4 h. Mitotic chromosome spreads were prepared, and kinetochore localization of Myc-Mps1 was detected by immunofluorescent staining with antibodies against the Myc-tag. As shown in Figure 4B, both Myc-Mps1-wt and the mutant counterpart Myc-Mps1-mut showed clear kinetochore localization; however, the signal intensity was stronger in cells expressing Myc-Mps1-mut (Figure 4C).

### Mps1 SUMOylation regulates mitotic progression

**Figure 5:**
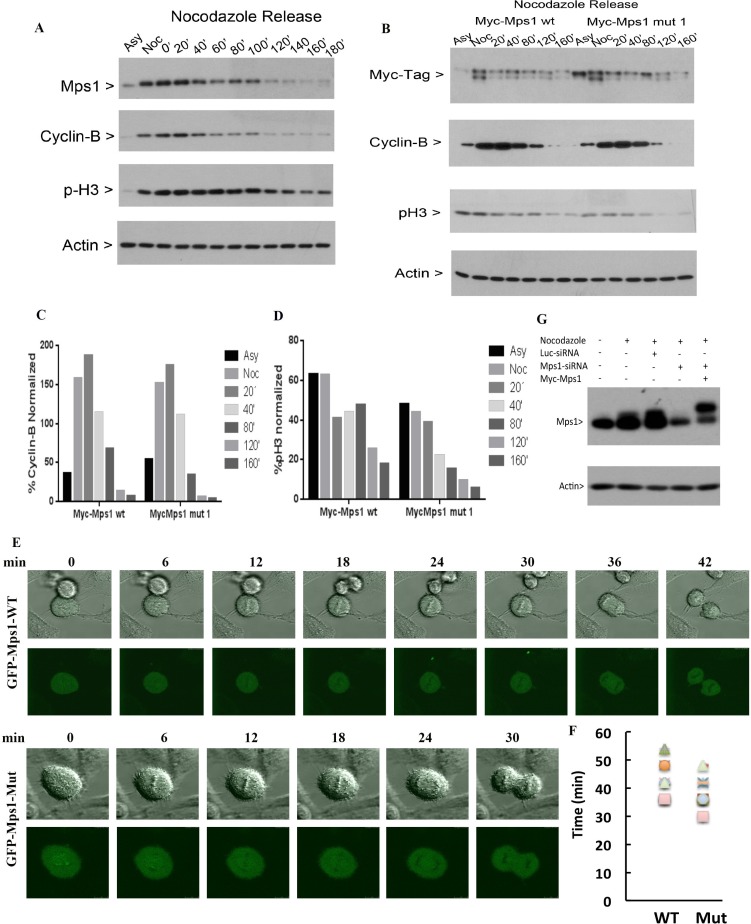
Sumoylation of Mps1 affects mitotic progression (**A**) HeLa cells were treated with nocodazole for 14 h. Round-up cells were harvested by physical shake-off and subject to mitotic release for various times. Equal amounts of cell lysates were blotted for Mps1, cyclin B1, phospho-histone H3S10 (p-H3) and β-actin. (**B**) HeLa cells were transfected with plasmids expressing Myc-Mps1-wt or Myc-Mps1-mut for 48 h followed by treatment with nocodazole for 14 h. Mitotic cells were then release into the cell cycle. Equal amounts of lysates were then blotted for indicated antibodies. (**C** and **D**) Quantitation of the degradation rates of cyclin B (C) and pH3 from (B). (**E**) HeLa cells were transfected with siRNAs specific to Mps1 and plasmids expressing GFP-Mps1-wt or GFP-Mps1-mut for 24 h followed by time-lapse confocal microscopy. Representative images from are shown. (**F**). Summary of data obtained from experiments shown in (E) to show mitotic duration of cells expressing either GFP-WT (*n* = 8) and GFP-5R (*n* = 8). (**G**) HeLa cells were transfected with either control luciferase siRNAs or siRNAs targeting *Mps1* 3′-UTR region together with plasmids expressing Myc-Mps1 for 24 hours, followed by the nocodazole treatment for 16 hours. Equal amount of cell lysates prepared in 8 M urea was blotted for Mps1 and actin.

We next determine whether sumoylation-resistant mutant of Mps1 would affect mitotic progression. HeLa cells were transfected with a plasmid construct expressing Myc-Mps1-wt or Myc-Mps1-mut for 48 h, followed by treatment with nocodazole for 14 h. Mitotic round-up cells were collected by physical shake-off and then subjected to mitotic release. Cells were collected at various time points after release and equal amounts of lysates were subjected to Western blot analysis. As shown in Figure 5A, Mps1 levels peaked at prometaphase/metaphase stages in control cells, after which it gradually decreased correlated with the decline of both cyclin B1 and phosphorylated histone H3 (p-H3S10) signals, indicating that Mps1 may also mediate the mitotic exit. Interestingly, compared to cells expressing Myc-Mps1-wt, disappearance of cyclin B1 and p-H3^S10^ accelerated in cells expressing Myc-Mps1-mut (Figure 5B, 5C and 5D), suggesting that sumoylation of Mps1 may be involved in mitotic timing. We next transfected HeLa cells with plasmids expressing GFP-Mps1-wt or GFP-Mps1-mut together with siRNAs specific to endogenous Mps1. Twenty-four hours after transfection, cells with GFP signals were subject to analysis using time-lapse confocal microscopy. As shown in Figure 5E and 5F, the mitotic time of cells expressing GFP-Mps1-mut was shorter than that of cells expressing GFP-Mps1-wt (37.50 ± 6.21 min. vs. 43.50 ± 7.69 min). These results further support the notion that sumoylation plays a role in regulating the mitotic function of Mps1. To confirm the specific knockdown of endogenous of Mps1 by siRNAs, HeLa cells were transfected with either control luciferase siRNAs or siRNAs targeting *Mps1* 3′-UTR region together with plasmids expressing Myc-Mps1 for 24 hours, followed by the nocodazole treatment for 16 hours. Equal amount of cell lysates was blotted for antibodies against Mps1 and actin. As shown in Figure 5G, endogenous Mps1 was greatly reduced in cells transfected with Mps1-specific siRNAs, and the expression of Myc-Mps1 was not affected. Notably, the slightly slower mobility shift in cells expressing Myc-Mps1 was caused by the Myc tag.

### SUMOylation regulates the interaction of Mps1 with BubR1

**Figure 6:**
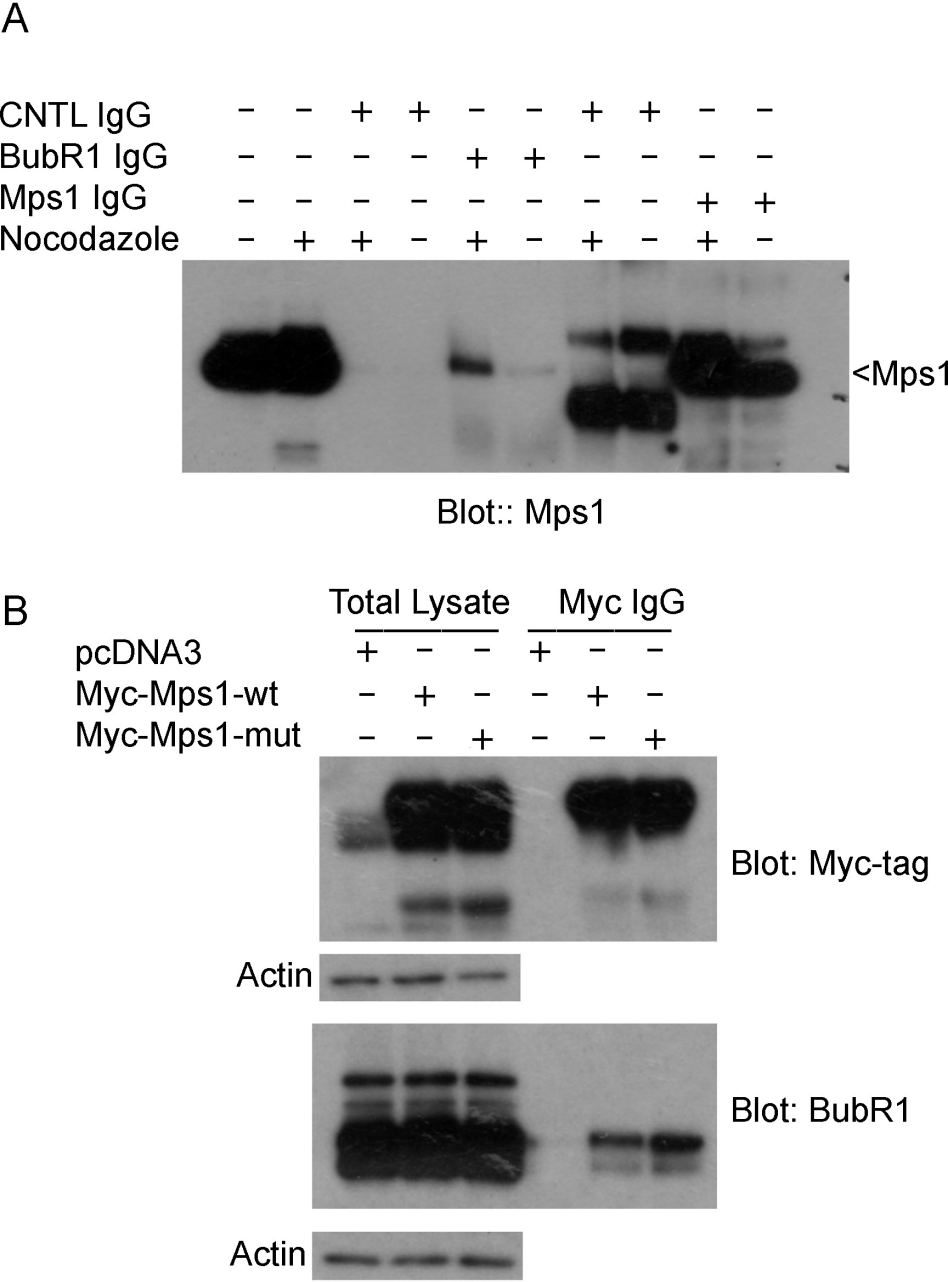
Mps1 sumoylation regulates its interaction with mitotic checkpoint protein BubR1 (**A**) HeLa cells were treated with or without nocodazole for 16 h after which cells were lysed in a native condition and immunoprecipitated with antibodies to BubR1 or Mps1. Control IgGs were also used as controls. Protein precipitates were then blotted for Mps1. (**B**) HeLa cells were transfected with Myc-Mps1-wt, Myc-Mps1-mut, or pcDNA3 vector for 48 h followed by treatment with nocodazole for 16 h. Total cell lysates prepared in a native condition were immunoprecipitated with the antibody to Myc. Protein precipitates were then blotted for Myc-tag and BubR1. Total lysates were also blotted for β-actin.

Mps1 is known to be the up-stream regulator of SAC [30]. We then asked whether sumoylation would affect the interaction between Mps1 and other SAC components. HeLa cells were either asynchronized or treated with nocodazole for 16 h. Equal amounts of total cell lysates were subject to immunoprecipitation with antibodies against BubR1 or Mps1, and then blotted for Mps1. As shown in Figure 6A, Mps1 was specifically precipitated by Mps1 antibody but not by the control IgG. Notably, Mps1 was also efficiently precipitated by BubR1, but not a control, antibody, strongly suggesting the physical interaction between Mps1 and BubR1. We next transfected HeLa cells for 48 h with plasmids expressing Myc-Mps1-wt, Myc-Mps1-mut, or the control plasmid. After treatment with nocodazole for another 16 h, equal amounts of cell lysates were immunoprecipitated with the antibodies against MYC tag and BubR1. As shown in Figure 6B, ectopically expressed Mps1 was efficiently expressed and precipitated by the anti-Myc antibody. BubR1 was also precipitated by the Myc antibody. Significantly, a greater amount of BubR1 was precipitated from cells expressing Myc-Mps1-mut than that of the cells expressing Myc-Mps1-wt, strongly suggesting that sumoylation may regulate the interaction of Mps1 with BubR1.

## DISCUSSION

In this study, we show evidence that Mps1 is modified by both SUMO-1 and SUMO-2 during the cell cycle and that this post-translational modification occurs on multiple lysine residues including K71, K287, K367 and K471. Our study also shows that sumoylation may negatively affect Mps1's kinetochore localization during mitosis. Sumoylation appears to decelerate mitotic progression as mitotic cells expressing sumoylation-resistant mutant of Mps1 exit from mitosis faster than those expressing wild-type Mps1. Moreover, sumoylation regulates the physical interaction of Mps1 with BubR1 during mitosis, suggesting its possible role in SAC regulation.

The N-terminus of Mps1 is important for its nuclear translocation and kinetochore localization [9, 21, 31–35]. As all four lysine residues identified for SUMO modification reside in the N-terminal region of Mps1 we speculated that this modification may play a significant role in its subcellular localization. Intriguingly, protein fractionation of asynchronized cells did not reveal a major difference between cells expressing GFP-Mps1-wt and its SUMO-resistant mutant counterpart. On the other hand, Myc-Mps1-mut was enriched at the kinetochores of mitotic chromosomes more than it did with wild-type counterpart (Figure 4B). Moreover, when SAC was activated by nocodazole, enhanced chromatin binding was observed in cells expressing SUMO-resistant mutants (Figure 4A), suggesting that sumoylation may play a role in modulating kinetochore localization of Mps1. Consistent with this notion, many studies have shown that sumoylation is an important mechanism by which mitotic components assemble a functional kinetochore during mitosis [36–38].

As an Mps1 target, BubR1 plays critical roles in regulating SAC and mitotic timing. Physical interaction between Mps1 and BubR1 is observed in *Drosophila* [39]. However, it has not observed in mammalian cells [40]. In our current study, we demonstrated that endogenous Mps1 was precipitated by a BubR1-specific antibody, indicating their interaction. Endogenous BubR1 was also precipitated by the Myc-tag antibody in cells ectopically expressing Myc-Mps1. Combined, these results support the conclusion that Mps1 physically interacts with BubR1 *in vivo* in mammalian cells. Although the molecular mechanism remains unclear, sumoylation appears to negatively regulate the association of Mps1 with BubR1 in mitosis because increased level of BubR1 was pulled-down by Myc-Mps1-mut than that by Myc-Mps1-wt. In other words, the interaction between Mps1 and BubR1 may be fine-tuned through post-translational SUMO modification. Moreover, these observations also suggest that BubR1 kinetochore localization may be mediated, at least partially, through Mps1 sumoylation. Supporting this, it has been shown that although Mps1 is not required for the recruitment of BubR1 to unattached kinetochores [9] its absence increases the rate of BubR1 exchange on the kinetochores [39].

Mps1 primarily functions as a mitotic kinase [31, 32]. Although identified four lysine residues essential for sumoylation reside in the N-terminus, we are not sure whether sumoylation will change the overall protein structure, consequently influencing the kinase activity towards its targets including BubR1. Given that inhibition of Mps1 kinase activity leads to enhanced kinetochore localization [11, 15, 41], we speculate that sumoylation will positively influence Mps1 kinase activity as cells expressing SUMO-resistant Mps1 display an increase level of kinetochore localization (Figure 4B).

Sumoylation regulates mitotic progression via monitoring chromosome congression, chromosome alignment, and anaphase onset [38, 42]. Depletion of Mps1 by siRNA causes dysfunction of kinetochores along with premature mitotic exit. In our study, cells expressing GFP-Mps1-mut coupled with depletion of endogenous Mps1 exhibited a shortened mitotic duration than those expressing GFP-Mps1-wt (Figure 5). Intriguingly, we did not observe increased premature chromosome separation, suggesting partial rescuing of premature chromosome segregation. These results also suggest that SUMO-deficient mutants fail to fully rescue impaired SAC function due to Mps1 depletion. Since cells that are lack of functional Mps1 become aneuploid and subsequently die [43, 44] it will be of great interest to know whether daughter cells that express GFP-Mps1-mut display increased chromosome missegregation.

It has also been shown that Mps1 plays a role in regulating centrosome function [10, 45–48]. In our current study, we did not see clear centrosome localization of ectopically expressed GFP-Mps1 or GFP-Mps1-mut in interphase cells, which is likely due to the fact that only a small proportion of Mps1 localizes to the centrosome. Interestingly, when cells expressing GFP-Mps1-mut or GFP-Mps1-wt were treated with nocodazole followed by the release into the cell cycle, a higher proportion of mutant-expressing cells displayed aberrant centrosome structures (e.g., tri-centrosomes) than those expressing GFP-Mps1-wt (data not shown). Thus, sumoylation may mediate Mps1's centrosomal function during mitosis.

## MATERIALS AND METHODS

### Cell culture and transfection

HeLa and HEK293T cell lines were obtained from the American Type Culture Collection. HeLa cells stably expressing His6-SUMO-1 or His6-SUMO-2 were kindly provided by R.T. Hay [23]. Cells were cultured in DMEM supplemented with 10% fetal bovine serum (FBS, Invitrogen) and antibiotics (100 μg/ml of penicillin and 50 μg/ml of streptomycin sulfate, Invitrogen) at 37°C under 5% CO_2_. The stable cell lines were maintained under 2 mM of puromycin (Sigma-aldrich).

Transfection of plasmids or siRNAs was achieved with either Fugene HD (Roche Diagnostic) or Lipofectamine 2000 (Invitrogen) following the manufacturer's instructions. Mitotic shake-off cells were obtained from gentle tapping of cells treated with nocodazole or taxol (50 ng/ml) (Sigma-Aldrich) for 14 h. Both types of shake-off cells were used for mitotic release in the presence or absence of nocodazole as specified.

### Antibodies

Rabbit polyclonal antibodies for BubR1 were developed in the laboratory. Antibodies for Myc-Tag, p-H3S10, Cyclin B1, and β-actin were purchased from Cell Signaling Technology Inc. Mouse monoclonal antibody for Mps1 was purchased from Invitrogen. GFP antibodies were purchased from Santa Cruz Biotechnology. Mouse anti-FLAG and anti-α-Tubulin were purchased from Sigma Aldrich. Mouse anti-SUMO-1 and SUMO-2/3 antibodies were kindly provided by Dr. Michael J. Matunis (John Hopkins University). Human IgGs (CREST) against centromere proteins were purchased from Antibodies Incorporated (Davis, CA).

### Plasmids, mutagenesis, and siRNAs

Various Mps1 mutants with lysine residues (K71, K287, K336, K367, K471 and/or K772) replaced with arginines (R) were generated using the QuickChange Lightning Multi Site-directed Mutagenesis kit (Stratagene). Individual mutations were confirmed by DNA sequencing (Seqwright). His6-SUMO-1 plasmid was purchased from Addgene. FLAG-SENP-1 and its mutant expression plasmids were kindly provided by J. Cheng [24]. UBC9 cDNA was subcloned into a FLAG-expression vector. Synthetic siRNA specific to the Mps1 gene 3′-untranslated region (5′-UUG-CUA-UCC-ACC-CAC-UAU-UUU-3′), as well as the control siRNA, were purchased from Dharmacon RNAi Technology.

### Western blot

SDS-PAGE was carried out using the mini-gel system from Bio-Rad. Proteins were transferred to PVDF (Polyvinylidene fluoride) membranes. After blocking with TBST buffer (50 mM Tris, 150 mM NaCl, 0.05% Tween 20) containing 5% nonfat dry milk for 1 h, the membranes were incubated overnight with primary antibodies, followed by incubation with horse-radish peroxidase-conjugated secondary antibodies (Cell Signaling Technology) for 1 h at room temperature. After thorough washing with TBST buffer, signals were developed with an enhanced chemiluminescent system (Pierce).

### Immunoprecipitation and pull-down assays

For immunoprecipitation, cells were lysed in a lysis buffer (20 mM Tris, pH 7.5, 150 mM NaCl, 1% Triton, 2 mM sodium pyrophosphate and 1 mM EDTA, 1 mM NaF, 1 mM sodium orthovanadate, 500 mM PMSF, 2 mM pepstatin A, 10 units/ml aprotinin, 20 mM NEM) and cleared by centrifugation. 1 μg of antibody and 25 μl of protein G-agarose resin (50/50, Millipore) were then added to 2 mg cell lysates and incubated at 4°C overnight followed by five washing with the lysis buffer. Proteins bound to resin were eluted with SDS sample buffer and then subject to analysis by SDS-PAGE followed by Western blot with appropriate antibodies.

For pull-down assays under denaturing conditions, cells were lysed in a lysis buffer (8 M urea, 50 mM Na_2_HPO_4_/NaH_2_PO_4_ (pH 7.4), 300 mM NaCl, 0.1% Triton X-100) containing 20 mM imidazole. Ni-IDA Superflow Resin (Clontech) was added to the cell lysates and incubated with gentle agitation at room temperature for 2 h. The resin was then washed 3 times at room temperature with the lysis buffer containing 40 mM imidazole. After the last wash, His6-tagged proteins were eluted in the lysis buffer containing 300 mM imidazole and blotted for Mps1, the MYC-tag, SUMO-1, or SUMO-2.

### Protein fractionation

Cells washed twice with cold PBS were lysed in buffer 1 (10 mM Tris-HCl pH 7.9, 10 mM KCl, 1.5 mM MgCl_2_, 1 mM DTT) and left on ice for 10 min. After centrifugation (2000 × g × 10 min) the supernatant (the cytoplasmic extract) were collected and the pellet fraction was re-suspendend in buffer 2 (20 mM Tris-HCl pH 7.9, 1.2 mM KCl, 1.5 mM MgCl_2_, 25% (w/v) glycerol, 0.2 mM EDTA) followed by gentle agitation for 1 h at 4°C. The supernatants (the nucleoplasmic/loose chromatin binding fraction) were harvested after centrifugation (16,000 g × 15 min). The pellets (chromatin binding) were re-suspended in a lysis buffer (8 M urea, 50 mM Na_2_HPO_4_/NaH_2_PO_4_ (pH 7.4), 300 mM NaCl, 0.1% Triton X-100).

### Mitotic chromosome spreads and fluorescence microscopy

To obtain mitotic chromosome spreads, cells transfected with various expression constructs were treated with 40 ng/mL nocodazole for 4 h and then collected after Trypsin-EDTA treatment and incubated in 40% PBS at 37°C for 15 min. Cells were resuspended in a fixative solution (glacial acetic acid/methanol in a ratio of 1:3) prior to spreading onto microscope slides (Fisher Scientific). Chromosome spreads were incubated overnight with antibodies to Myc-tag and CREST. After incubation with Alex Fluor 488-conjugated goat anti-mouse IgG and Alex Fluor 555-conjugated goat anti-human IgG, cells were counterstained with 4, 6-diamidino-2-phenylindole (DAPI, Molecular Probe, Eugene, OR). Chromosomal images were captured with a Leica TCS SP5 confocal microscope or a Leica AF6000 fluorescence microscope.

For time-lapse video imaging, HeLa cells seeded onto chamber slides (Lab-TeK) were transfected with various expression constructs for 24 h. The transfected cells were then cultured in a humidified chamber at 37°C in CO_2_-independent medium (GIBCO-BRL). GFP-positive cells were subject to time-lapse video imaging on a Leica TCS SP5 confocal microscope.
